# Topological alterations in white matter anatomical networks in cervical dystonia

**DOI:** 10.1186/s12883-024-03682-4

**Published:** 2024-05-27

**Authors:** Jiana Zhang, Yuhan Luo, Linchang Zhong, Huiming Liu, Zhengkun Yang, Ai Weng, Yue Zhang, Weixi Zhang, Zhicong Yan, Jinping Xu, Gang Liu, Kangqiang Peng, Zilin Ou

**Affiliations:** 1 Department of Neurology, The First Affiliated Hospital, Guangdong Provincial Key Laboratory for Diagnosis and Treatment of Major Neurological Diseases, National Key Clinical Department and Key Discipline of Neurology, Sun Yat-sen University, Guangzhou, 510080 China; 2 Department of Medical Imaging, State Key Laboratory of Oncology in Southern China, Collaborative Innovation Center for Cancer Medicine, Sun Yat-Sen University Cancer Center, Guangzhou, 510060 China; 3 Institute of Biomedical and Health Engineering, Shenzhen Institutes of Advanced Technology, Chinese Academy of Sciences, Shenzhen, 518055 China

**Keywords:** Cervical dystonia, Clinical manifestations, Cortico-subcortical white matter, Diffusion tensor imaging, Graph theoretical analysis

## Abstract

**Background:**

Accumulating neuroimaging evidence indicates that patients with cervical dystonia (CD) have changes in the cortico-subcortical white matter (WM) bundle. However, whether these patients’ WM structural networks undergo reorganization remains largely unclear. We aimed to investigate topological changes in large-scale WM structural networks in patients with CD compared to healthy controls (HCs), and explore the network changes associated with clinical manifestations.

**Methods:**

Diffusion tensor imaging (DTI) was conducted in 30 patients with CD and 30 HCs, and WM network construction was based on the BNA-246 atlas and deterministic tractography. Based on the graph theoretical analysis, global and local topological properties were calculated and compared between patients with CD and HCs. Then, the AAL-90 atlas was used for the reproducibility analyses. In addition, the relationship between abnormal topological properties and clinical characteristics was analyzed.

**Results:**

Compared with HCs, patients with CD showed changes in network segregation and resilience, characterized by increased local efficiency and assortativity, respectively. In addition, a significant decrease of network strength was also found in patients with CD relative to HCs. Validation analyses using the AAL-90 atlas similarly showed increased assortativity and network strength in patients with CD. No significant correlations were found between altered network properties and clinical characteristics in patients with CD.

**Conclusion:**

Our findings show that reorganization of the large-scale WM structural network exists in patients with CD. However, this reorganization is attributed to dystonia-specific abnormalities or hyperkinetic movements that need further identification.

## Background

Idiopathic cervical dystonia (CD) is the most frequent form of local dystonia and is characterized by abnormal head, neck and shoulder movements and postures [1–3]. In addition, CD is associated with tremors and pain, together with motor manifestations impair daily living activities and decrease the quality of life [4, 5]. However, the underlying causes and pathophysiology of CD remain poorly understood.

Accumulating evidence from functional magnetic resonance imaging (MRI) and voxel-based morphometry demonstrates the occurrence of functional and structural abnormalities in multiple brain regions [6–9], including the basal ganglia, thalamus, cerebellum, and cerebral cortex, showing a network model in which various brain regions play a role in the CD pathogenesis. In addition, Ramdhani et al. [10], found that gray matter alterations were accompanied by more widely observed white matter (WM) microstructural abnormalities in patients without task-specific dystonia (blepharospasm and CD) than in those with task-specific dystonia (writer’s cramp and laryngeal dystonia). These abnormalities were located mainly in the brainstem, thalamus, corpus callosum, anterior limb/genu of the internal capsule, cerebellum, primary sensorimotor cortex, WM of the middle/inferior frontal gyrus, and inferior temporal gyrus. In patients with CD, diffusion tensor imaging (DTI) studies using a region of interest-based analysis or a whole-brain approach have detected extensive WM microstructural changes in the motor cortex, premotor cortex, frontal, temporal and parietal cortices, visual system, basal ganglia, thalamus, cerebellum, and brainstem [8, 11]. In addition, DTI studies using tractography-based method demonstrated abnormal connections between the pallidum and brainstem [12], the dentato-rubro-thalamic tract, between the thalamus, middle frontal gyrus and brainstem [13], and between the globus pallidus, putamen, thalamus and the sensorimotor cortices [14]. Therefore, observing abnormalities in DTI studies indicates that diffuse and extensive loss of WM integrity may be a common feature of CD. Our previous study investigating topological changes in WM structural networks using graph theory analysis in patients with blepharospasm found that these patients display large-scale WM reorganization in the brain at the network level [15]. However, studies revealing the overall connection changes in WM anatomical networks in CD, rather than those observed in specific anatomical structures, are still lacking. Graph theoretical methods model the brain as a complex network whose topological architecture can be quantitatively characterized. Following the network modeling procedure, various network properties can be employed to investigate the organizational mechanisms underlying the relevant networks. This approach provides tools for understanding the association between various pathological processes and diseases [16, 17]. Therefore, a detailed knowledge of large-scale WM anatomical network reorganization can help researchers better understand the pathophysiological mechanisms underlying CD and facilitate the development of therapeutic strategies.

In this study, we hypothesized that extensive reorganization of large-scale WM anatomical networks occurs in patients with CD. We tested this hypothesis by combining DTI with graph theoretical analysis to study the differences in topological organization between CD patients and healthy controls (HCs). In addition, the relationships between the identified topological metrics and clinical features (e.g., symptom severity and disability) were further evaluated in patients with CD.

## Methods

### Participants

Patients with idiopathic CD, and HCs were recruited from our outpatient movement disorder clinic between September 2021 and July 2023. All patients met the following inclusion criteria: (i) age 19–75 years; and (ii) a diagnosis of CD was established according to the published criteria by an experienced neurologist (G.L.) [18]. Exclusion criteria were as follows: (i) known causes of dystonia or a family history of movement disorders; (ii) had dystonia involving other body sites in addition to neck muscles; (iii) reported evidence of Parkinson’s disease, stroke, traumatic brain injury, Alzheimer’s disease, and epilepsy; (iv) had a family history of movement disorders as well as a history of exposure to antipsychotic drugs before the onset of dystonia; (v) had any conditions that contradicted with cerebral MRI; (vi) received botulinum toxin (BoNT) injections within 3 months and/or oral medications for approximately 24 h before MRI scans. HCs were recruited using the same exclusion criteria. Written informed consent was obtained from all the participants and the study was approved by the Ethical Committee of the First Affiliated Hospital of Sun Yat-sen University ([2020]323).

## Clinical assessment

Demographics and clinical characteristics, including patients’ age, sex, duration of disease, and number of BoNT injections were collected from all patients via face-to-face interviews before MRI scanning. The Toronto Western Spasmodic Torticollis Rating Scale (TWSTRS), Global Dystonia Rating Scale (GDS), and Cervical Dystonia Impact Profile-58 (CDIP-58) were used to assess the severity, disability, and effects of CD on the quality of life [19–21]. Non-motor symptoms, including anxiety, depression, and cognition situation were assessed using the Hamilton Anxiety Scale (HAMA) [22], Hamilton Depression Scale (HAMD) [23], and Minimum Mental State Examination (MMSE) [24], respectively.

### Data acquisition

MRI data for each participant were collected using a 3T scanner (Tim Trio; Siemens, Erlangen, Germany). DTI data were acquired using a single-shot echo-planar imaging sequence (repetition time, 7000 ms; echo time, 91 ms; flip angle, 90°; acquisition matrix, 128 × 128; field of view, 256 × 256 mm^2^; voxel size, 2 × 2 × 3 mm^3^; 50 axial slices). Diffusion weighting was isotropically distributed in 64 directions using a b value of 1000 s/mm^2^. Moreover, three dimensional T1-weighted images (repetition time = 2530 ms, echo time = 4.45 ms, inversion time = 1100 ms, flip angle = 7°, matrix dimensions = 256 mm × 256 mm, voxel size = 1 × 1 × 1 mm^3^, and 192 slices) were obtained to improve registration to the standard space.

### Image preprocessing

All DTI data were analyzed using the PANDA toolbox (Pipeline for Analyzing Brain Diffusion Images toolkit, https://www.nitrc.org/projects/panda/) with structural MRI of the brain [25]. First, the brain mask was estimated for each participant from the b0 image, and the non-brain tissues were removed. Subsequently, the data were corrected for head motion and eddy current distortion by applying the affine registration of each diffusion-weighted image to the b0 image. The diffusion tensor elements were then estimated, and the fractional anisotropy (FA) was calculated for each voxel. The generated FA images were registered to the Montreal Neurological Institute (MNI) 152 standard space using nonlinear registration. Subsequently, the whole-brain fiber tractography was performed by a deterministic tracking algorithm using the Diffusion Toolkit (http://trackvis.org) and TrackVis software (http://trackvis.org/) [26]. All tracts were computed by seeding each voxel with an FA > 0.2. Tractography was terminated if it turned at an angle exceeding 45° or reached a voxel with an FA of less than 0.2.

### Brain network construction

**Fig. 1:**
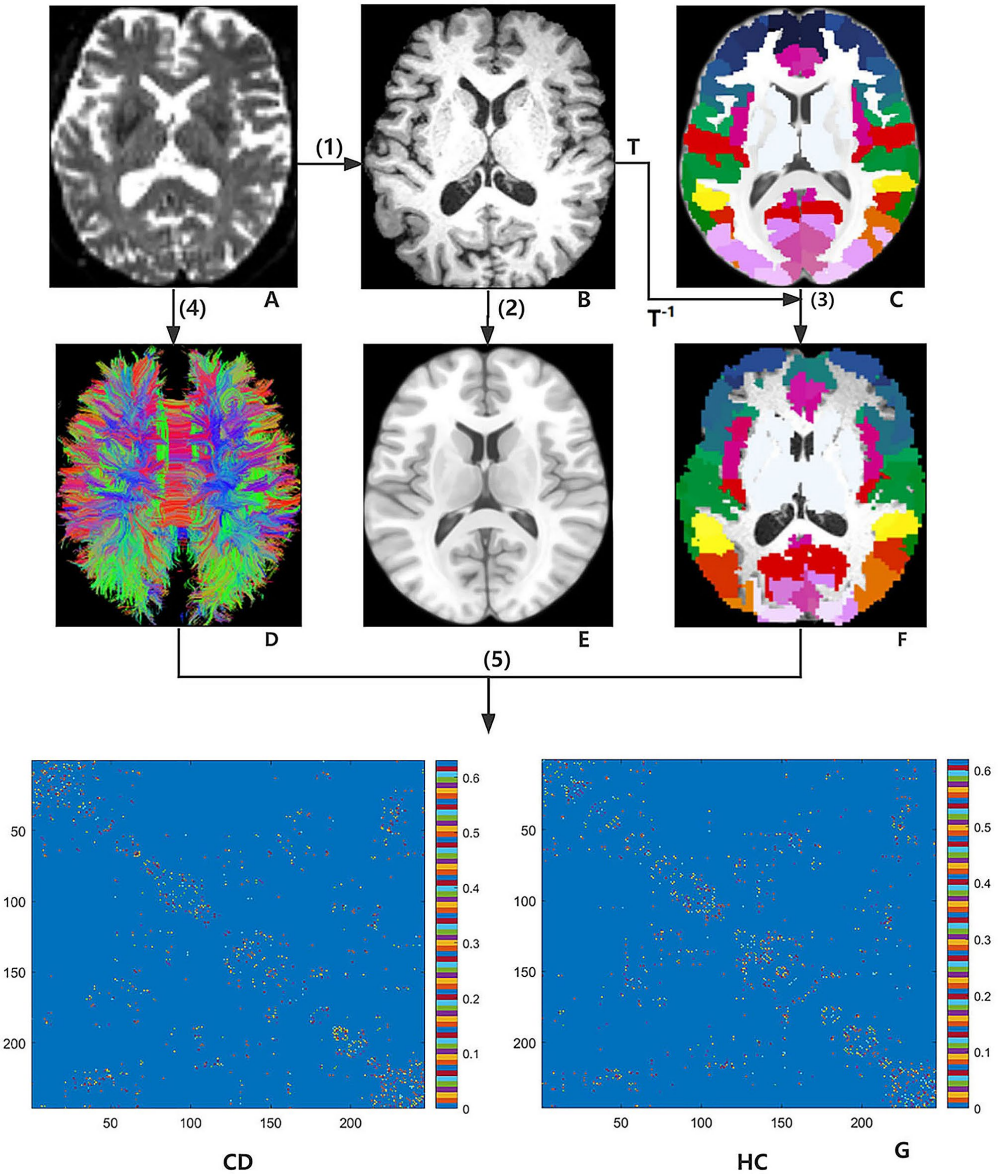
Flow diagram of WM network construction applying DTI based on BNA-246 atlas. (1) b0 image (**A**) in the individual diffusion space for each participant was aligned with the T1-weighted image (**B**). (2) The co-registered T1-weighted image of the native DTI space was mapped to the nonlinear ICBM152 T1 template (**E**) of the MNI space using a nonlinear transformation matrix T. (3) Warped the BNA-246 atlas (**C**) in MNI space into the native diffusion space (**F**) by inverting T1 format T^− 1^. (4) Whole-brain WM fibers (**D**) were traced with DTI tractography employing the deterministic tractography algorithm. (5) The whole brain WM network was constructed in patients with CD and HCs. FSe-weighted matrices (**G**) for CD and HCs groups. Abbreviations: BNA-246, Brainnetome Atlas with 246 brain regions; CD, cervical dystonia; DTI, diffusion tensor imaging; HCs, healthy controls; ICMB, International Consortium for Brain Mapping; MNI, Montreal Neurological Institute; WM, white matter

Adopting the approach used in our previous study (Guo et al., 2021) [15], network nodes were defined using the BNA-246 atlas (http://atlas.brainnetome.org/). This fine-grained parcellation atlas includes more detailed information on both functional and anatomical connections, which could help to describe connectivity in the brain global network characteristics more accurately [27]. We set the fraction of streamline (FSe) values as the edge weights of the network [28]. Reportedly, FSe was implemented to estimate the strength/weight of each WM connection in tractography-derived matrices [28]. The FSe value for a pathway originating in some Brainnetome region A and terminate at B is defined as the ratio of the number of streamlines originating at A and terminating at B to the total number of streamlines that either originate at A or terminate at B while excluding streamlines that represent self (within-area) connections [29]. As a connectivity matrix of 246 × 246 regions, the FSe links two regions relative to the number of streamlines extrinsic to those regions. Finally, a symmetric FSe-weighted (246 × 246) matrix, representing the WM structural network, was generated for each participant (Fig. 1) [29].

$$FSe(i,j)=\frac{NOS(i,j)}{{\sum }_{x=1}^{N}NOS(i,x)+\sum _{y=1}^{N}NOS(y,j)-NOS(i,j) }$$

where χ ≠ i and y ≠ j; N is the total number of regions (*N* = 246); and NOS (i, j) is the number of streamlines connecting regions i and j.

### Global network measures

On the global level, the integration (e.g., global efficiency [*E*_*glob*_], shortest path length [*L*_*p*_]), segregation (local efficiency [*E*_*loc*_], cluster coefficient [*C*_*p*_], and modularity [*Q*]), and resilience (assortativity [*r*]) measures [30] of WM anatomical networks were computed based on the FSe-weighted (246 × 246) matrix for each participant by using the Gretna toolbox (https://helab.bnu.edu.cn/gretna/) [31], which was implemented using the MATLAB (R2018b) platform (https://www.mathworks.com/). The network density, also belonging to the integration of the network [32], was calculated by invoking the network density function in the Brain Connectivity Toolbox (https://www.nitrc.org/projects/bct/). In addition, network strength (*S*_*p*_) and small-world properties were assessed to characterize the topological organization of the networks. *E*_*glob*_ can indicate the efficiency of information transference across a network, while *E*_*loc*_ indicates how well a node exchanges information with its neighboring nodes [33]. A smaller *L*_*p*_ indicates the faster information transfer to the entire brain region, and *C*_*p*_ quantifies the prevalence of clustered connectivity around individual nodes [34]. Network density refers to the ratio of actual to possible connections [30]. Additionally, assortativity, known as degree correlation, is a measure of the correlation between nodal degree and mean degree of its nearest neighbors, which is related to the more vulnerable network being attacked by lower assortativity [35–37]. Positive assortativity values indicate that nodes may be connected to other nodes of the same degree, and high-degree nodes or hubs of the network are likely to be connected [30]. A small-world network is defined as γ > 1 and λ ≈ 1. These two measurements can be summarized into a simple quantitative metric, small worldness, σ = γ/λ > 1 [38].

### Regional network measures

We calculated the nodal efficiency (*E*_*nodal*_) and two metrics of nodal centrality, betweenness centrality (BC) and degree centrality (DC), for all 246 regions [39]. *E*_*nodal*_ represents the importance of a given node in conveying information within a network. BC is the fraction of all shortest paths in the network that passes through a given node. The DC, the number of links connected to a node, is one of the most common centrality measures [40].

### Statistical analyses

Differences in age between patients with CD and HCs were assessed using two sample *t*-tests or Mann-Whitney U tests after Shapiro-Wilk normality testing. Sex distributions were compared using the Pearson χ^2^ test. All analyses were performed using SPSS (version 27.0; IBM, Armonk, NY, USA).

Between-group differences in streamline count and network parameters were determined using two sample t-test, with HAMA and HAMD scores as covariates. In addition, the relationships between the abnormal network graph theoretical metrics and clinical features, including TWSTRS total scores, TWSTRS subscales, and disease duration, were assessed using partial correlation analyses after adjusting for age, sex, HAMA, and HAMD scores. *P* < 0.05 was set to evaluate statistical significance.

### Reproducibility analyses

Referring to the previous study [15], we evaluated the potential effects of different parcellation schemes employing similar network analyses with an additional Anatomical Automatic Labeling atlas with 90 brain regions (AAL-90; https://www.gin.cnrs.fr/en/tools/aal/) in patients with CD [41].

## Results

### Demographic information and clinical characteristics

The demographic and clinical characteristics of the 30 patients with CD (14 women; median age, 40 years) and 30HCs (16 women; median age, 41 years) are summarized in Table 1. There were no statistically significant differences in age, sex, MMSE scores, or streamline count between the groups. A significant difference in the HAMA and HAMD scores between the groups was observed. We visually inspected the tractography to ensure a necessary minimum coverage of the track count including the corpus callosum (1161), cingulate (240), uncinate tracts (222), superior (370) and inferior longitudinal fasciculus (208), corticospinal tract (2683), and spinothalamic tract (2438) in patients with CD.

**Table 1:** Demographics and clinical characteristics of the participants

	CD (*n* = 30)	HCs (*n* = 30)	*P* value
Median age (range)	40 (19–70)	41 (23–62)	0.549
Female (%)	14 (46.7%)	16 (53.3%)	0.606
Median duration year (range)	1.25 (0.12-10)	-	-
BoNT injections (yes/no)	9/21	-	-
Median TWSTRS (range)	33.75 (15.75–56.50)	-	-
Median TWSTRS severity (range)	19 (7–25)	-	-
Median TWSTRS disability (range)	10 (0–23)	-	-
Median TWSTRS pain (range)	5.5 (0-10.5)	-	-
Median GDS (range)	6 (1–18)	-	-
Median CDIP-58 (range)	48.97 (16.90–73.10)	-	-
Median HAMA (range)	7 (0–23)	1 (0–8)	< 0.001
Median HAMD (range)	6 (0–18)	1 (0–7)	< 0.001
MMSE (range)	28 (25–30)	29 (25–30)	0.121
Streamline count (Mean ± SD)	42296.5 ± 5360.3	42370.6 ± 6180.8	0.961

Abbreviations: CD, cervical dystonia; CDIP-58, Cervical Dystonia Impact Profile; GDS, Global Dystonia Rating Scale; HAMA, Hamilton Anxiety Scale; HAMD, Hamilton Depression Scale; HCs, healthy controls; MMSE, Minimum Mental State Examination; TWSTRS, Toronto Western Spasmodic Torticollis Rating Scale

### Differences in global and regional network properties

**Fig. 2:**
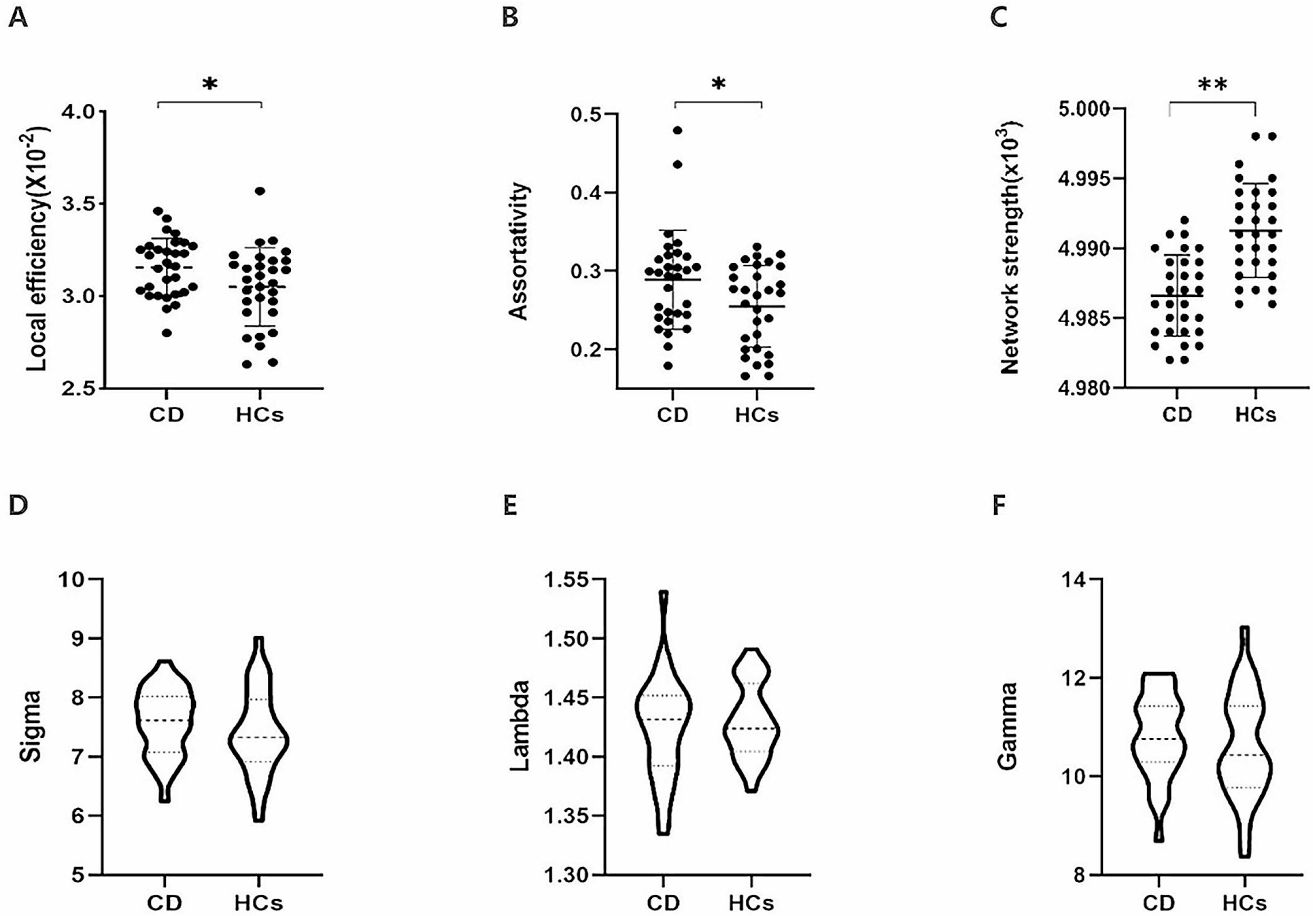
Characteristics of global properties between patients with CD and HCs by BNA-246 atlas. The global parameters of local efficiency (**A**), assortativity (**B**) and network strength (**C**) had shown significant differences between CD and HCs. Small-world properties (D, E and F) were displayed both CD and HCs (Sigma > 1, Lambda ≈ 1 and Gamma > 1). *P < 0.05, **P < 0.001, compared with HCs. Abbreviations: BNA-246, Brainnetome Atlas with 246 brain regions; CD, cervical dystonia; HCs, healthy controls

Compared with HCs, a significant increase of *E*_*loc*_ (*t* = 2.231, *P* = 0.029), and assortativity (*t =* 2.264, *P* = 0.027), and a significant decrease of *S*_*p*_ (*t* = 5.349, *P* < 0.001) were found in patients with CD (Table 2; Fig. 2A , B, and C). However, no significant differences in *E*_*glob*_, *C*_*p*_, *L*_*p*,_, *Q*, and network density were observed in these patients. Patients with CD and HCs showed a small world architecture of the WM network characterized by γ > 1 and λ ≈ 1 (Fig. 2D, E, and F). Patients with CD showed higher *L*_*p*_ and lower *E*_*glob*_ than HCs, but the differences were not statistically significant. In addition, we did not find any differences in the regional network properties such as *E*_*nodal*_, BC, or DC between patients with CD and HCs.

**Table 2:** Differences of global properties between CD patients and HCs by BNA-246 and AAL-90 atlas

		CD (*n* = 30)	HCs (*n* = 30)	*P* value
BNA-246	Local efficiency	0.032 ± 0.002	0.031 ± 0.002	0.029 *
	Assortativity	0.289 ± 0.063	0.255 ± 0.052	0.027 *
	Network strength	4986.60 ± 2.90	4990.60 ± 2.90	< 0.001**
	Network density	0.056 ± 0.002	0.055 ± 0.001	0.669
	Shortest path length	58.267 ± 0.410	58.191 ± 0.303	0.883
	Global efficiency	0.01718 ± 0.0001	0.01720 ± 0.0008	0.928
AAL-90	Assortativity	0.131 ± 0.069	0.083 ± 0.061	0.029 *
	Network strength	6018.60 ± 2.90	6022.60 ± 2.90	< 0.001 **
	Network density	0.273 ± 0.003	0.272 ± 0.004	0.812
	Shortest path length	39.230 ± 0.229	39.113 ± 0.253	0.768
	Global efficiency	0.0255 ± 0.0001	0.0256 ± 0.0001	0.782

Abbreviations: AAL-90, Anatomical Automatic Labeling atlas with 90 brain regions; BNA-246, Brainnetome Atlas with 246 brain regions; CD, cervical dystonia; HCs, healthy controls. *Note* *P < 0.05, **P < 0.001, compared with HCs.

### Correlations between network metrics and clinical features

No significant correlations were found between altered network properties and clinical characteristics, including TWSTRS total scales, TWSTRS subscales, disease duration.

### Reproducibility of results

We repeated the reconstruction of the WM network using the AAL-90 atlas. Similar results characterized by higher assortativity (*t* = 2.237, *P* = 0.029) and lower *S*_*p*_ (*t* = 5.349, *P* < 0.001), were observed in patients with CD than in HCs. However, differences in *E*_*loc*_ between patients with CD and HCs could not be reproduced. We also found no differences in nodal network metrics such as *E*_*nodal*_, BC, and DC, between patients with CD and HCs.

## Discussion

In this study, we found that patients with CD displayed alterations in segregation characterized by increased *E*_*loc*_ and resilience and increased assortativity in the whole-brain WM anatomical brain network. These findings support our hypothesis that extensive reorganization of large-scale WM anatomical networks occurs in patients with CD.

Neuroimaging studies have demonstrated the small-world characteristics of large-scale WM networks in the human brain [42, 43]. Consistently, both patients with CD and HCs showed typical small-world WM network topologies. In addition, the findings of our previous study revealed a small-world architecture of the WM network in patients with blepharospasm [15]. This evidence supports the perspective that the structural architecture of the human brain is conserved and that small-world networks can tolerate disease and developmental alterations [44]. Local efficiency, an index of structural segregation, is associated with short range connections between neighborhood regions, which increase the fault tolerance of a network or mediate modularized information processing [33]. Higher local efficiency in patients with CD indicates dense local connections in brain structural networks [45]. Assortativity represents the degree of correlation between connected nodes. High assortativity, termed assortative mixing, indicates that nodes of a similar degree tend to be connected in the network [46, 47]. Generally, an assortative-mixing network can accelerate the spread of information generated by high-degree nodes [35, 48]. A positive assortativity coefficient implies that nodes with similar degrees are more likely to connect with each other, indicating enhanced local information processing efficiency and resilience to attack [49], while increased local efficiency implies closed local connections [45]. Moreover, increased *L*_*p*_ and decreased *E*_*glob*_ have been suggested to be associated with a reduced transmission and integration function of long-distance brain interval information in post-traumatic stress disorder and type 2 diabetes [50, 51]. Thus, the positive and higher assortativity and *E*_*loc*_, together with the increased *L*_*p*_ and decreased *E*_*glob*_ values in patients with CD, indicate enhanced local interconnectivity and local information processing abilities that might be associated with the loss of long-distance brain interval information communication. However, there were no significant differences in *L*_*p*_ and *E*_*glob*_ between patients with CD and HCs, possibly due to the small sample size of this study. Further analyses with larger sample sizes are needed to clarify these differences. Additionally, structural connectivity tending to form an assortative network for a connected network reportedly may help to keep the flow of neural signals and processing as far as possible, while functional connectivity reversely form a dissortativity network to assist functional connectivity maintaining the functionality against indiscriminate propagation of disturbance [52]. The assortativity of functional networks in patients with CD should be further verified in future studies.

However, we could not determine whether changes in *E*_*loc*_ and assortativity were attributable to the dystonia-specific reorganization of structural brain networks or to cervical hyperkinetic movements. A lack of association between increased *E*_*loc*_ and assortativity and symptom severity of CD demonstrates that these changes are more likely because of dystonia origin. However, further studies are needed to confirm this. In this study, we did not find any differences in regional network properties such as *E*_*nodal*_, BC, and DC in patients with CD compared to HCs, which is not consistent with the findings of our previous study involving in patients with blepharospasm [15]. In our previous study, patients with blepharospasm showed increased nodal efficiency values in multiple cortical and subcortical regions, including the primary motor cortex and basal ganglia, compared with HCs. These areas are hub regions specific to blepharospasm. Lesion studies have revealed that damage to the cerebellum and the nearby brainstem is more likely to result in CD [3, 53]. In addition, Prudente et al. [54] investigated the neuropathology of cervical dystonia in six patients and found a patchy loss of Purkinje cells in the cerebellum. Recent animal and clinical studies have shown that dystonia is closely associated with abnormalities in the motor network involving the cerebellum and dystonia [55, 56]. This evidence shows that network changes in the cerebellum may have a closer relationship with CD, considering the absence of infratentorial brain structures such as the cerebellum and brainstem in the BNA-246 atlas. However, further studies are needed to address this hypothesis.

Validation analyses showed increased assortativity significantly and *S*_*p*_ were similarly detected using the AAL-90 atlas in patients with CD compared with HCs. However, we also noted inconsistent findings. For example, no differences in local efficiency observed between patients with CD and HCs. A possible explanation for this inconsistency is that the local *E*_*loc*_ is more sensitive to selecting parcelllation atlases than other global topological metrics.

This study had some limitations. First, the sample size of this study was relatively small. Second, the current understanding of the effects of BoNT on WM structural networks is limited, highlighting the need for further research in this area. Third, the absence of the brainstem and cerebellum in the BNA-246 atlas, limits exploring the roles of network changes in these regions in CD pathophysiology and their correlation with disease severity. Fourth, DTI deterministic tractography was used to reconstruct human WM anatomical networks. Although this method widely used, its ability to resolve crossed fiber bundles is limited [57]. Therefore, probabilistic diffusion tractography approaches should be used in further studies to reconstruct brain WM networks.

In conclusion, our findings demonstrate that patients with CD exhibit widespread brain reorganization at the network level. Detailed knowledge of large-scale WM anatomical network reorganization can help researchers better understand pathophysiological mechanisms of CD, However, whether this reorganization is attributed to dystonia-specific abnormalities or hyperkinetic movements in cervical muscles needs further identification.

## Data Availability

The data and materials supporting the findings of this study are available from the corresponding author upon reasonable request.

## Abbreviations

AAL-90: Anatomical Automatic Labeling atlas with 90 brain regions
BC: Betweenness Centrality
BNA-246: Brainnetome Atlas with 246 brain regions
BoNT: Botulinum Toxin
CD: Cervical Dystonia
DC: Degree Centrality
DTI: Diffusion Tensor Imaginge
FA: Fractional Anisotropy
GDS: Global Dystonia Rating Scale
HAMA: Hamilton Anxiety Scale
HAMD: Hamilton Depression Scale
RM ANOVA: Repeated measures analysis of variance
HCs: Healthy Controls
MMSE: Minimum Mental State Examination
MNI: Magnetic Resonance Imaging
MNI: The Montreal Neurological Institute
TWSTRS: Toronto Western Spasmodic Torticollis Rating Scale

## References

[1] Claypool DW, Duane DD, Ilstrup DM (1995). Epidemiology and outcome of cervical dystonia (spasmodic torticollis) in Rochester, Minnesota. Mov Disord.

[2] Fahn S, Bressman SB, Marsden CD (1998). Classification of dystonia. Adv Neurol.

[3] LeDoux MS, Brady KA (2003). Secondary cervical dystonia associated with structural lesions of the central nervous system. Mov Disord.

[4] Rosales RL, Cuffe L, Regnault B (2021). Pain in cervical dystonia: mechanisms, assessment and treatment. Expert Rev Neurother.

[5] Maione R, Formica C, Quartarone A (2023). The impact of non-motor symptoms on quality of life in cervical dystonia. J Clin Med.

[6] Battistella G, Termsarasab P, Ramdhani RA (2017). Isolated focal Dystonia as a disorder of large-scale functional networks. Cereb Cortex.

[7] Norris SA, Morris AE, Campbell MC (2020). Regional, not global, functional connectivity contributes to isolated focal dystonia. Neurology.

[8] Pontillo G, Castagna A, Vola EA (2020). The cerebellum in idiopathic cervical dystonia: a specific pattern of structural abnormalities?. Parkinsonism Relat Disord.

[9] Giannì C, Pasqua G, Ferrazzano G (2022). Focal dystonia: functional connectivity changes in cerebellar-basal ganglia-cortical circuit and preserved global functional architecture. Neurology.

[10] Ramdhani RA, Kumar V, Velickovic M (2014). What’s special about task in dystonia? A voxel-based morphometry and diffusion weighted imaging study. Mov Disord.

[11] Prell T, Peschel T, Köhler B (2013). Structural brain abnormalities in cervical dystonia. BMC Neurosci.

[12] Blood AJ, Kuster JK, Woodman SC (2012). Evidence for altered basal ganglia-brainstem connections in cervical dystonia. PLoS ONE.

[13] Sondergaard RE, Rockel CP, Cortese F (2021). Microstructural abnormalities of the dentatorubrothalamic tract in cervical dystonia. Mov Disord.

[14] Giannì C, Piervincenzi C, Belvisi D (2023). Cortico-subcortical white matter bundle changes in cervical dystonia and blepharospasm. Biomedicines.

[15] Guo Y, Peng K, Liu Y (2021). Topological alterations in white matter structural networks in blepharospasm. Mov Disord.

[16] Bassett DS, Bullmore ET (2009). Human brain networks in health and disease. Curr Opin Neurol.

[17] He Y, Chen Z, Gong G (2009). Neuronal networks in Alzheimer’s disease. Neuroscientist.

[18] Albanese A, Bhatia KP, Cardoso F (2023). Isolated cervical dystonia: diagnosis and classification. Mov Disord.

[19] Comella CL, Leurgans S, Wuu J (2003). Rating scales for dystonia: a multicenter assessment. Mov Disord.

[20] Cano SJ, Warner TT, Linacre JM (2004). Capturing the true burden of dystonia on patients: the cervical Dystonia Impact Profile (CDIP-58). Neurology.

[21] Comella CL, Perlmutter JS, Jinnah HA (2016). Clinimetric testing of the comprehensive cervical dystonia rating scale. Mov Disord.

[22] Hamilton M (1959). The assessment of anxiety states by rating. Br J Med Psychol.

[23] Hamilton M (1960). A rating scale for depression. J Neurol Neurosurg Psychiatry.

[24] Folstein MF, Folstein SE, McHugh PR (1975). Mini-mental state. A practical method for grading the cognitive state of patients for the clinician. J Psychiatr Res.

[25] Cui Z, Zhong S, Xu P (2013). PANDA: a pipeline toolbox for analyzing brain diffusion images. Front Hum Neurosci.

[26] Mori S, Crain BJ, Chacko VP (1999). Three-dimensional tracking of axonal projections in the brain by magnetic resonance imaging. Ann Neurol.

[27] Fan L, Li H, Zhuo J (2016). The human Brainnetome Atlas: a new brain atlas based on connectional architecture. Cereb Cortex.

[28] Donahue CJ, Sotiropoulos SN, Jbabdi S (2016). Using Diffusion Tractography to predict cortical connection Strength and Distance: a quantitative comparison with tracers in the monkey. J Neurosci.

[29] Zhao C, Yang L, Xie S (2019). Hemispheric Module-Specific influence of the X chromosome on White Matter Connectivity: evidence from girls with Turner Syndrome. Cereb Cortex.

[30] Rubinov M, Sporns O (2010). Complex network measures of brain connectivity: uses and interpretations. NeuroImage.

[31] Wang J, Wang X, Xia M (2015). GRETNA: a graph theoretical network analysis toolbox for imaging connectomics. Front Hum Neurosci.

[32] Boroda E, Armstrong M, Gilmore CS (2021). Network topology changes in chronic mild traumatic brain injury (mTBI). Neuroimage Clin.

[33] Latora V, Marchiori M (2001). Efficient behavior of small-world networks. Phys Rev Lett.

[34] Olde Dubbelink KT, Hillebrand A, Stoffers D (2014). Disrupted brain network topology in Parkinson’s disease: a longitudinal magnetoencephalography study. Brain.

[35] Newman ME (2002). Assortative mixing in networks. Phys Rev Lett.

[36] Sun J, Bagrow JP, Bollt EM (2009). Dynamic computation of network statistics via updating schema. Phys Rev E Stat Nonlin Soft Matter Phys.

[37] Williams O, Del Genio CI (2014). Degree correlations in directed scale-free networks. PLoS ONE.

[38] Watts DJ, Strogatz SH (1998). Collective dynamics of ‘small-world’ networks. Nature.

[39] van den Heuvel MP, Sporns O (2013). Network hubs in the human brain. Trends Cogn Sci.

[40] Mukhtar MF, Abal Abas Z, Baharuddin AS (2023). Integrating local and global information to identify influential nodes in complex networks. Sci Rep.

[41] Zhao K, Zheng Q, Che T (2021). Regional radiomics similarity networks (R2SNs) in the human brain: reproducibility, small-world properties and a biological basis. Netw Neurosci.

[42] Hagmann P, Kurant M, Gigandet X (2007). Mapping human whole-brain structural networks with diffusion MRI. PLoS ONE.

[43] Iturria-Medina Y, Canales-Rodríguez EJ, Melie-García L (2007). Characterizing brain anatomical connections using diffusion weighted MRI and graph theory. NeuroImage.

[44] Achard S, Bullmore E (2007). Efficiency and cost of economical brain functional networks. PLoS Comput Biol.

[45] Micheloyannis S, Pachou E, Stam CJ (2006). Small-world networks and disturbed functional connectivity in schizophrenia. Schizophr Res.

[46] Brandes U (2008). On variants of shortest-path betweenness centrality and their generic computation. Soc Networks.

[47] Murakami M, Ishikura S, Kominami D (2017). Robustness and efficiency in interconnected networks with changes in network assortativity. Appl Netw Sci.

[48] D’Agostino G, Scala A, Zlatić V (2012). Robustness and assortativity for diffusion-like processes in scale-free networks. EPL.

[49] Sun M, Xie H, Tang Y (2020). Directed network defects in Alzheimer’s disease using granger causality and graph theory. Curr Alzheimer Res.

[50] Lei D, Li K, Li L (2015). Disrupted functional brain connectome in patients with posttraumatic stress disorder. Radiology.

[51] Xu J, Chen F, Liu T (2019). Brain functional networks in type 2 diabetes Mellitus patients: a resting-state functional MRI study. Front Neurosci.

[52] Lim S, Radicchi F, van den Heuvel MP (2019). Discordant attributes of structural and functional brain connectivity in a two-layer multiplex network. Sci Rep.

[53] Jinnah HA, DeFazio G (2023). Adult-onset focal dystonias: to lump or split. Int Rev Neurobiol.

[54] Prudente CN, Pardo CA, Xiao J (2013). Neuropathology of cervical dystonia. Exp Neurol.

[55] Prudente CN, Hess EJ, Jinnah HA (2014). Dystonia as a network disorder: what is the role of the cerebellum?. Neuroscience.

[56] Shakkottai VG, Batla A, Bhatia K (2017). Current opinions and areas of Consensus on the role of the Cerebellum in Dystonia. Cerebellum.

[57] Mori S, van Zijl PC (2002). Fiber tracking: principles and strategies - a technical review. NMR Biomed.

